# Delays in Cryptococcal Meningitis Diagnosis and Care: A Mixed Methods Study in Rural Uganda

**DOI:** 10.5334/aogh.3524

**Published:** 2022-03-18

**Authors:** Abigail Link, Mark Okwir, Betty Nabongo, David Meya, Sarah Iribarren, Paul Bohjanen, Danuta Kasprzyk

**Affiliations:** 1 University of Minnesota, US; 2 Lira University, UG; 3 Lira Regional Referral Hosptial, UG; 4 Makerere University, UG; 5 University of Washington, US

## Abstract

**Background:**

Cryptococcal meningitis (CM) remains a major cause of mortality for HIV-infected persons in sub-Saharan Africa, despite widespread access to antiretroviral therapy. Delays in CM diagnosis and treatment contribute to high mortality, with patients often arriving “too late” for treatment to be effective. Little is known about patient-related delays and their experiences with CM.

**Objectives:**

This study seeks to identify the factors related to delays in diagnosis and care among patients with cryptococcal meningitis.

**Methods:**

A convergent mixed-methods approach was used to understand delays related to diagnosis and treatment of CM among patients admitted to Lira Regional Referral Hospital in rural northern Uganda. We collected data from February to March 2020 using surveys followed by semi-structured interviews from 20 CM patients who survived hospitalization and 20 family members of deceased patients during February 2017–November 2019. Interviews were audio-recorded, transcribed, and thematically coded for analysis.

**Findings:**

Delays to CM care were related to 1) self-medication, 2) lack of CM education, 3) seeking treatment multiple times at health centers with 4) missed/misdiagnosis, and 5) cultural factors. Among patients who died, 70% sought care ≥3 times, while those who survived, 35% of sought care ≥3 times before CM diagnosis. Only 10% of patients and 40% of family members knew what caused CM, indicating a lack of knowledge.

**Conclusions:**

Patients sought medical care for CM symptoms, but several factors contributed to CM diagnosis and care delays. Many of these factors relate to a lack of CM education and knowledge among patients and providers. A CM awareness campaign for the general public, targeted education for HIV patients, and continuing medical education for healthcare providers can decrease delays and improve outcomes.

## Introduction

Cryptococcal meningitis (CM) affects 220 000 people worldwide [1] with an estimated mortality rate at 50–70% [2]. It continues to be one of the most common killers of people with HIV/AIDS [3] and is still occurring in the era of accessible antiretroviral therapy (ART). Immunosuppression related to ART treatment failure or non-adherence has been identified as a factor for continued CM occurrence [4,5]; however, other factors involved in CM care-seeking behavior are unknown. Studies attributed high mortality among CM patients to delays in care [2,6,7], specifically patients arriving “too late” for CM diagnosis and treatment [6,8,9]. Other studies suggested delays in CM diagnosis were related to limited resources for diagnosis and treatment, lack of provider awareness, and limited staff training for complex medical management [2,10,11,12]. Recommendations to improve these delays focused on logistical interventions, such as providing adequate supplies for CM treatment and diagnostics [2,8,13], and early detection through screening. [7]. These interventions; however, do not address the reasons why patients present late for CM care, when treatment is less successful, and outcomes become dire. Delays in CM diagnosis and treatment lead to poor outcomes and the reasons for these delays from the patient’s perspective are poorly understood. This study seeks to identify and understand the reasons for delays in order to make informed recommendations to improve CM outcomes.

## Methods

### Study design

A convergent mixed-methods design was used to evaluate patients and family members of patients hospitalized with CM from February 2017– February 2019 at Lira Regional Referral Hospital (LRRH) in Uganda. Patient and family member surveys were conducted then immediately followed by semi-structured interviews from February to March 2020. Semi-structured interviews allowed for deeper probing of issues of interest after the survey, which provided more robust understanding of the survey responses through the incorporation of the participant’s contextual perspective and experience [14].

### Procedures

Twenty patients diagnosed with CM at LRRH, aged ≥13, who provided consent and/or assent were sampled in order to directly obtain patient perspectives. Additionally, 20 family members of deceased CM patients, aged ≥ 18, who were knowledgeable about the patient and were caretakers while they were hospitalized at LRRH, were sampled to understand the experiences of patients who died. All eligible patients who were diagnosed or family members of patients with CM from February 2017– February 2019 and had available contact information, were purposively sampled. Phone numbers were located through patient records or clinical record forms. Participants were invited by telephone to participate, with some patients approached at follow-up appointments. To reach our sample size, patients and family members were invited on an ongoing basis after February 2019, until the sample size was reached in November 2019. If patients died after discharge and a family member who attended to the patient was available, they were also invited to participate.

Surveys were conducted at LRRH and proctored by trained interviewers in Lango or English. The surveys were completed on an electronic tablet using REDCap (Research Electronic Data Capture), a secure, HIPPA compliant, web-based application [15]. Interviews were audio recorded, transcribed, then translated into English (when required). To verify the validity of the translation, audits of the transcriptions were assessed by each transcriptionist to ensure accuracy of the transcription. Transcripts were entered into ATLAS.ti for analysis [16].

### Theoretical model

**Figure 1:**
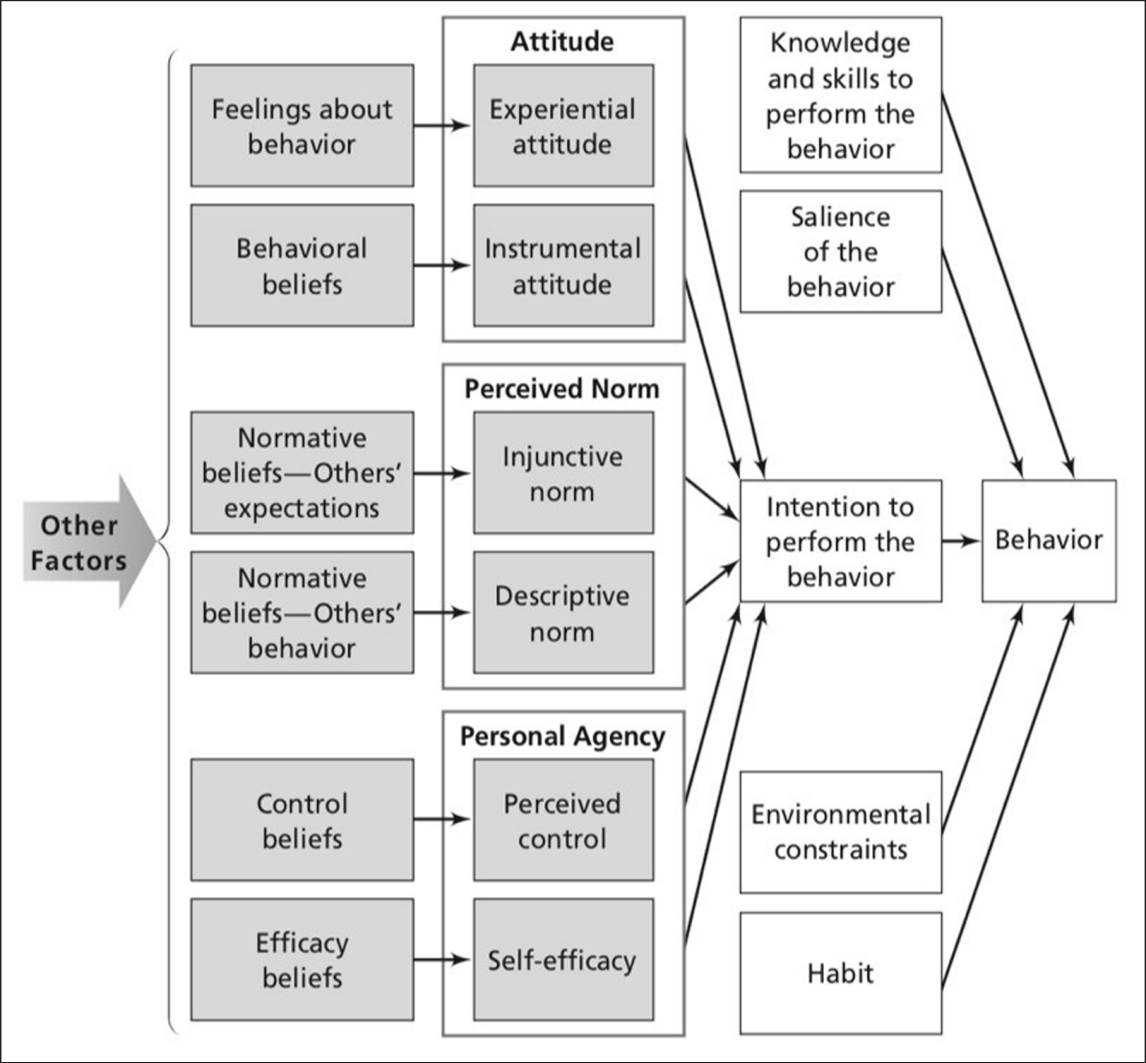
Integrated Behavioral Model.

Questions from the survey and interviews used the Integrated Behavioral Model (IBM) as a framework for assessing what factors impact a patient’s decision-making in terms of their health-seeking behaviors (***Figure 1***). This model assumes that the best predictor of performing a behavior is linked to intention or motivation [17] and incorporates attitude, perceived norms and personal agency with additional factors that may affect behavioral intention such as the environment, habit, knowledge, and salience of the behavior. This study utilized the constructs from this model to formulate questions for the surveys and semi-structured interviews. Incorporating this model to our study provided a greater understanding of the gaps and drivers for behaviors related to care-seeking and treatment in patients.

### Analyses

Quantitative data was analyzed through descriptive statistics, using means and proportions for the responses from the surveys regarding demographic information and barriers to care-seeking behavior, diagnosis and treatment. Qualitative analysis was conducted using content analysis, through open coding and memo writing using the IBM framework to guide the codes. From these codes, networks and memos were created to generate further codes and identification of major themes related to patient emotions and behavior towards ART, HIV diagnosis, stigma, and delays in care and treatment for HIV and CM. A primary code book was established to provide continuity in coding between coders. Additional codes were added based on participants’ responses. All coded interviews were reviewed to ensure accuracy and validity and to minimize discrepancies in coding between transcripts. Triangulation between qualitative and quantitative data were explored to identify areas of convergent and divergent responses.

### Ethics and Consent

This study obtained approval from Gulu University Research Ethics Committee [GUREC 107–19], Uganda National Council of Science and Technology [SS 5151], University of Washington Institutional Review Board (IRB) [STUDY00007770]. All participants signed an informed consent and/or assent form before the surveys and interviews began.

## Results

### Demographic and clinical data for patients with CM who survived or died

We compared demographic information for those who lived or died, based on our survey data. The average age between those who lived and died was 39.3 and 34.9 years, respectively, revealing that those who died were slightly younger (***Table 1***). The groups had similar education status, with 30–40% having secondary education or higher. Marital status was the same between the groups and those who survived had a higher average monthly income compared to those who died at ($50.49 vs. $43.85, exchange rate: 3700 Uganda shillings to 1 USD). Overall, the demographic information was similar between those who lived or died.

**Table 1:** Demographic Information.


DEMOGRAPHICS	EXPIRED PATIENT	SURVIVING PATIENT

Age (mean, range)	34.9, 13–60	39.3, 17–63

Sex: Female (N, %)	10, 50	6, 30

Marital Status: Married (N, %)	10, 50	10, 50

Employment status	**(N, %)**	**(N, %)**

Full time	7, 35	7, 35

Part-time	0	3, 15

Seasonal	3, 15	2, 10

Unemployed	10, 50	8, 40

Education		

Post-secondary	3, 15	4, 20

Secondary	4, 20	4, 20

Primary	12, 60	9, 45

None	1, 5	1, 5

Average Monthly Income (USD currency)	(Mean, Range)	(Mean, Range)

	$43.85 ($0.03–140.5)	$50.49 ($2.70–172.73)


### Care-seeking behaviors in response to symptoms

The most common symptoms patients experienced were headache (90%) followed by fever (42.5%) and neck pain (27.5%; ***Table 2***). The majority of patients self-medicated (85%) for the primary purpose of pain relief (55%). The average waiting period from the time of symptom onset to arrival at LRRH and CM diagnosis was 163 days for patients who died and 15.4 days for patients who survived. A total of 97.5% of patients received treatment at a health facility prior to their admission to LRRH. Among those who died, 70%, sought care ≥3 times in comparison to among those who survived, of which only 35% sought care ≥3 times. These data suggest that patients had a prolonged period of meningitis symptoms, tried to self-medicate, and sought care multiple times before a diagnosis of CM was made. Patients who died had a longer duration of symptoms and sought medical care more often.

**Table 2:** Health-Seeking Behavior.


	EXPIRED PATIENT (N, %)	SURVIVING PATIENT (N, %)

**Symptoms that prompted health seeking**		

Headache	19, 95	17, 85

Fever	9, 45	9, 45

Neck pain	4, 20	8, 40

**Treatment sought at other health facility**		

Yes	19, 95	20, 100

**Type of health facility**		

Clinic/Health Center II	3, 15	7, 35

Health Center III	8, 40	7, 35

Hospital/Health Center IV	7, 35	4, 20

LRRH	2, 10	4, 20

**Times seen at other health facility prior to LRRH**		

None (directly to LRRH)	1, 5	1, 5

1	2, 10	7, 35

2	2, 10	5, 25

≥3	14, 70	7, 35

**Reason for health care treatment**		

Worse symptom, no improvement	16, 80	14, 70

Referred	2, 10	2, 10

Seeking meds	2, 10	3, 15

HIV meds	1, 5	2, 10

**Diagnosis at health facility**		

Malaria	7, 35	8, 40

HIV	6, 30	2, 10

Typhoid	4, 20	0

Meningitis	0	2, 10

CM	2, 10	1, 5

**Days waited until treatment sought**	(Mean, Range)	(Mean, Range)

Days	163, 2–1095	15.4, 1–60


### Knowledge about CM

Results from the survey found that less than half the patients and family members had heard of CM prior to their diagnosis (35%), and fewer patients knew that it was recurrent compared to family members (20% vs. 50%; ***Table 3***). Only 10% of patients and 40% of family members knew that CM was caused by fungus, while 17.5% of patients and family members knew all the complications of CM.

**Table 3:** CM Knowledge.


KNOWLEDGE	FAMILY MEMBER	PATIENT

HEARD OF CM PRIOR TO DIAGNOSIS	(N, %)	(N, %)

Yes	7, 35	7, 35

**CM can be recurrent**		

Yes*	10, 50	4, 20

**CM Cause**		

Fungus*	8, 40	2, 10

Bacteria, Virus, or Tuberculosis	8, 40	12, 60

Unknown	4, 20	6, 30

**Complications of CM**		

Vision loss	5, 25	5, 25

Hearing loss	1, 5	0

Death	8, 40	7, 35

All the above*	3, 15	4, 20

I don’t know	3, 15	4, 20


* Denotes correct answer.

### Behaviors contributing to delays in care

Semi-structured interviews provided further information regarding reasons for delay in the diagnosis and treatment of CM. The initial symptoms of CM, such as headache or fever, are non-specific and present similarly to common diseases such as typhoid or malaria. Ninety percent of family members and 80% of patients shared that they experienced missed opportunities for earlier CM diagnosis and care because of potential delays through self-medication, multiple primary health center visits, missed/misdiagnosis, or cultural/religious beliefs.

#### Self-medication leading to delays in care

We found that most patients self-medicated prior to seeking medical care; often for pain relief. Most patients attempted self-medication at home (87.5%) prior to LRRH admission. Some patients had symptoms and self-medicated for several months before they sought medical care. The following statements highlight some of the reasons for delay related to patient’s believing their symptoms were mild enough to self-medicate and treat at home:

The reason why she took long without treatment because she was taking painkiller that would sometimes make her feel better. (Family Member of 41-year-old female patient)

She was taking drugs, which were bought from clinics or drug shops. Sometimes she buys only Panadol and then takes. (Family Member of 33-year-old female patient)

When patients felt they improved with self-medication, this perpetuated delays in medical care.

#### Seeking care at multiple health facilities led to delayed CM diagnosis

Most patients (n = 39 of 40) sought care at another health facility before they were diagnosed with CM at LRRH. Going to multiple health centers, where a diagnosis of CM was missed, resulted in patients growing weaker and led to further delay in CM diagnosis and treatment. One family member shared their experience:

We moved in different health centers like Aber health center, Abongomola Health Center III, when we saw no improvement, [we] decided to come here to the main hospital [LRRH]. (Family Member of 29-year-old male patient)

Most participants started their care from smaller health centers because they were closer to their homes and thought they could be managed from there. Thus, LRRH became the last stop for health care through referral or because they wanted a higher level of care.

### The role of missed diagnoses or misdiagnoses in delays in care

Patients sought care at multiple medical facilities prior to their admission to LRRH, without obtaining a CM diagnosis. If a correct diagnosis was made, the number of visits and delays would have been minimized. This highlights issues of inadequate diagnostic testing and knowledge on the part of health care providers. Some patients were not tested but were symptomatically diagnosed (potential misdiagnosis) and prescribed medications. Others were tested and diagnosed with diseases such as malaria or typhoid and were treated with little improvement. Some of the patient’s accounts support these types of delays:

I only went to the clinic. They didn’t test me, but they only gave medicines. But those medicines didn’t reduce it. (Patient, F, age 31)

We were talking of malaria, typhoid, but after treatment both diseases we found that the sickness was still… persisting, and it was hard for us to manage, for them [health center] to manage. (Family Member of 30-year-old female patient)

Once patients presented to LRRH, they were tested and treated for CM through an ongoing CM treatment program. However, delays could have been avoided if CM was suspected and testing was completed at their local health facilities.

### Influence of culture on delays to care

A strong sense of cultural or traditional beliefs are held and practiced among Ugandans, and some of these beliefs can conflict with information viewed through a westernized medical lens. As a result, many Ugandans continue to seek care from traditional and religious healers.

#### Traditional medicine and witch doctors

Seeking guidance and treatment through traditional healers is a common practice, especially among those living in rural areas. People seek traditional healers to perform ceremonies to bring rain during the dry season, curse or remove a curse from an adversary, or obtain traditional herbs and medicines for their illnesses. Some of these beliefs and traditions are engrained within the Ugandan culture and some patients embrace these practices. Perspectives on these practices are conveyed in the following:

I kept taking traditional medicine and then it [symptoms] disappeared, then in November, I went somewhere, and it came back. (Patient, M, age 34)

…a sister of mine there told me that it’s possible someone has bewitched you, they should first take me to someone [witch doctor] there to see…I refused. It was of benefit [because] if I had gone there, I would have been dead already… (Patient, male, age 48)

Patients seeking care from traditional healers can lead to delays in CM diagnosis and care because patients believe they are being healed and may feel that they do not need further care. Approximately 7.5% of the participants sought care from traditional healers or witch doctors prior to their CM diagnosis. However, this number may be underestimated as this information was unsolicited and was unveiled organically throughout the interviews.

#### Religion

Religion also plays an important part of Ugandan’s lives as 84.5% practice Christianity [18]. These beliefs of healing powers through prayer or religious rituals can dissuade patients from continuing medication or seeking medical care or treatments in hopes that prayer will cure their HIV or other illnesses. The following quotes demonstrate this:

…she started telling me ‘I went to TB Joshua (pastor in Nigeria) to pray for me’… I told her, ‘You my sister, you are not taking your medication right,’ and she kept quiet. That is when she told me… ‘They told me to stop taking drugs.’ (Family Member of 40-year-old female patient)

Some evils also added to it [symptoms] and then they took me for prayers, then they prayed and found other things, they prayed. Then the part for prayers ended, they said, take him to the hospital. Then they took me to the hospital. (Patient, M, age 14)

Despite the conflicts between cultural and traditional beliefs with practices of western medicine, some patients were able to integrate both practices without feelings of mutual exclusivity, leveraging the support that both practices bring to promote health and healing. Nonetheless, reports from participants suggested that religious beliefs contributed to delays in care as 7.5% sought prayers prior to their CM diagnosis.

### Lack of CM education and knowledge

All patients and family members reported some education related to HIV, but few stated they had education about CM. More than half the participants (60%) had never heard of CM prior to their diagnosis and lacked education and knowledge about CM. Exemplars from participants supporting their lack of education related to CM prevention or treatment included, “What are the prevention measures [for CM]?” and “…there is no knowledge [about CM], I was just told to take drugs well,” which indicated that patients and family members did not receive education related to CM prevention or treatment. Compliance to take medications because “they were told to” was expressed among 25% of the patients, but they did not understand why they were taking the medications. CM knowledge was identified as the information patients were able to recall and verbalize when asked questions about CM. The level of understanding of CM was higher among family members, who had more correct responses to the knowledge questions from the survey, than patients (n=21 vs. 10). This may be attributed to family members being older or having past experience with CM through other friends or family members. Having no knowledge about CM contributed to their delays in seeking health care as patients were unaware of the risks or urgency of their symptoms. The following statements reinforced a lack of understanding about CM:

I only want to understand it [CM], and if it is coming, how does it begin? And if it is healing, what damage does it leave on a person? (Patient, M, age 17)

He [uncle] wanted us to start with mental health [psychiatric ward] first. It was body pain and the treatment I got from the mental health unit was not even helping me. (Patient, M, 59).

The lack of knowledge among patients and family members was supported by their comments surrounding their knowledge of CM were evident, identifying gaps in their education.

## Discussion

We identified several themes related to delays in CM diagnosis, treatment, and care among patients and family members. These themes focused on behaviors, culture, education, knowledge and external factors of missed/misdiagnosis. Most participants experienced multiple factors within each of these themes which attributed to delays in diagnosis and care. A major finding dispelled the assumption that patients are arriving too late at health facilities for diagnosis and treatment as most patients visited multiple health facilities prior to their diagnosis of CM at LRRH. The length of symptom days was markedly different between patients who lived and died. We reassessed this data through validation with the patient’s hospital chart data which found the average length of symptoms for both living and deceased patients was the same at 22.3 days. This showed that family members of deceased patients overestimated the patient’s symptom days while the patients underestimated their days of illness indicating recall bias for both groups. This finding also validates participants had an average of three weeks of symptoms before diagnosis with CM and supports delays to diagnosis and care.

### Delays related to multiple health facility visits and opportunities of missed/misdiagnosis

Ninety-five percent of the participants sought healthcare prior to LRRH admission and were seen multiple times at other health facilities. Among those who died, 70% sought care ≥3 times compared to 35% of those who survived showing a two-fold increase of clinic visits among those who died versus those who survived. Our data shows that multiple visits can increase delays to CM diagnosis and care leading to poorer outcomes for patients. Multiple visits without a CM diagnosis also support issues of misdiagnosis, or missed diagnosis of CM. It also highlights the lack of provider knowledge and awareness of CM symptoms, missing CM as a differential diagnosis. A series of case studies supports the lack of awareness and cognitive biases of healthcare providers when confronted with patients with CM symptoms [11,12]. Missed opportunities to diagnose, treat, and/or refer patients on their first visit creates delays to CM care, increases symptom duration and illness severity, which ultimately leads to preventable patient deaths.

A review of diagnostic errors reported that misdiagnosis can come from common conditions (like malaria), as well as rarer illnesses [19] such as CM. The most common diagnoses patients had from other health facilities before coming to LRRH were malaria (37.5%) and HIV (20%). Additionally, delays in referring to higher levels of care can also contribute to delays in diagnosis, with dire consequences for patients with CM.

### Lack of patient education, knowledge, and awareness

Patients and family members had little understanding of and received little education about CM. Many participants did not know the cause of CM or the possibility of reoccurrence if not treated properly. Moreover, many patients reported their lack of education on CM maintenance therapy. More education is needed for CM and must encompass information on the cause of CM, as well as its transmission, diagnosis, treatment, and prevention. CM symptoms can be similar to malaria or typhoid, leading to self-medication and delays to seeking medical treatment. This is consistent with findings from the Uganda National Health Survey (UNHS) regarding care-seeking behavior, which showed that the majority of Ugandans (57%) did not seek healthcare because they thought the illness was not severe enough for medical consultation [20]. The evident lack of information regarding the basic facts of CM could be alleviated through a CM awareness campaigns via television, radio, or newspapers. Disseminating information in this manner would provide an efficient means for rapid circulation to the larger community. Additionally, targeted patient education via counseling nurses and other clinical staff on common opportunistic infections such as tuberculosis and a CM should be implemented at the time of HIV diagnosis.

### Contribution of Culture on CM Delays

Other factors that contribute to delays are cultural factors: specifically, religious or traditional beliefs. Some patients delayed treatment in preference to seeking traditional treatments while others turned to religious beliefs and relied on prayers for healing. Because of these beliefs, some patients stopped all medications, including antiretroviral therapy for HIV, in the belief that they would be healed. This study found that 7.5% of the participants sought traditional medicines or witch doctors and 7.5% sought prayers or religious healing. However, this may be underestimated as we did not specifically ask about their experience with traditional medicines, witch doctors and religious practices. To bridge the exclusivity between these practices and western medicine, a partnership between traditional healers, religious leaders, and the medical community could help eliminate these conflicting situations. Providing awareness and education to the traditional and religious healers by creating collaborative versus juxtaposing positions can improve relationships between these communities, ultimately improving patient outcomes.

## Limitations

The use of telephone contacts from patient records to identify potential participants was a limitation in this study, as this excluded participants who did not have access to a telephone for financial or other reasons or did not provide a contact number during their hospitalization. Also, the sampling strategy introduced recall bias, as some patients or family members did not accurately recall some details, such as dates or days of symptoms. To mitigate this limitation, when conflicting information was given, data was cross-checked and validated with clinical records collected at the time of the participant’s hospitalization.

## Conclusion

Patients sought care for CM symptoms, but several factors contributed to patient delays in CM diagnosis and care. Specifically, patients’ care-seeking behavior at local health facilities multiple times, self-treatment, seeking alternative therapies, and local providers’ missed and/or misdiagnosis contributed to delays in CM diagnosis and care. These findings relate to the lack of CM education and knowledge among patients, traditional healers, and health providers in local primary care settings. Possible solutions to improve early diagnosis and management of CM include a CM campaign for the general public as a tool to increase awareness of CM. Additionally, we recommend continuing medical education for healthcare providers on CM causes, symptoms, diagnosis, and treatment. Lastly, providing targeted education to HIV-positive patients, religious and traditional healers focusing on symptoms of common opportunistic infections, including CM, will increase their awareness, likely alerting them to seek immediate medical care. Implementing these targeted education campaigns could support timely recognition of CM for patients, alternative healers, and providers, which could mitigate the current issues around delays to improve CM-related morbidity and mortality.
